# Cardiovascular Risk Factors in Patients with Addison's Disease: A Comparative Study of South African and Swedish Patients

**DOI:** 10.1371/journal.pone.0090768

**Published:** 2014-03-06

**Authors:** Ian Louis Ross, Ragnhildur Bergthorsdottir, Naomi Levitt, Joel Alex Dave, Desmond Schatz, David Marais, Gudmundur Johannsson

**Affiliations:** 1 Division of Endocrinology, Department of Medicine, University of Cape Town, Cape Town, South Africa; 2 Department of Endocrinology, Institute of Medicine, Sahlgrenska University Hospital, University of Gothenburg, Gothenburg, Sweden; 3 Department of Paediatrics, University of Florida, Gainesville, Florida, United States of America; 4 Division of Chemical Pathology, Clinical Laboratory Sciences, National Health Laboratory Service, University of Cape Town, Cape Town, South Africa; University of Leicester, United Kingdom

## Abstract

**Background:**

Patients with Addison's disease (AD) in Scandinavia have an increased risk for premature death due to cardiovascular disease (CVD). Serum lipids are important risk factors for CVD and vascular mortality. Replacement doses of hydrocortisone have historically been higher in Sweden than South Africa. The primary aim was to study the lipid profiles in a large group of patients with AD with the hypothesis that the lipid profile in patients in Sweden would be worse than in South Africa.

**Methods:**

In a cross-sectional study, 110 patients with AD (55 from South Africa, 55 from Sweden) matched for age, gender, ethnicity and BMI were studied. Anthropometric measures, blood pressure, lipids, highly sensitive C-reactive protein (hs-CRP) and adiponectin were studied.

**Results:**

All patients were Caucasian and the majority were women N = 36 (65.5%). Mean (standard deviation; SD) ages of the Swedish and South African patients were 52.9 (13.0) and 52.6 (14.4) years and BMI 25.3 (3.2) and 25.8 (4.1) kg/m^2^, respectively. The mean total daily hydrocortisone dose was greater in the Swedish patients than the South African patients, [33.0 (8.1) versus 24.3 (8.0) mg; p<0.0001]. South African patients had higher median (interquartilerange; IQR) triglycerides (TG) [1.59 (1.1–2.46) versus 0.96 (0.74–1.6) mmol/l; p<0.001], total cholesterol (TC) [6.02(1.50) versus 5.13 (0.87) mmol/l; p<0.001], LDL-C [4.43 (1.44) versus 2.75 (0.80) mmol/l; p<0.001] and median hs-CRP [2.15 (0.93–5.45) versus 0.99 (0.57–2.10) mg/L; p<0.003] and lower HDL-C [0.80 (0.40) versus 1.86 (0.46) mmol/l; p<0.001] than the Swedish patients. Approximately 20% of the patients in both cohorts had hypertension and diabetes mellitus.

**Conclusions:**

South African patients with AD have worse lipid profiles and higher hs-CRP compared to their matched Swedish patients, despite lower doses of hydrocortisone. It is uncertain at this time whether these are due to genetic or environmental factors.

## Introduction

There is a more than two-fold increase in relative risk of death in Swedish patients with Addison's disease (AD), compared to the background population, predominantly due to cardiovascular disease (CVD) [1], [2], with the greatest number of deaths from ischaemic heart disease followed by cerebrovascular disease [1]. In a diverse population of South African patients with AD, we previously demonstrated that AD patients had a wide range of cardiovascular risk factors including elevated triglycerides, lower high density lipoprotein cholesterol (HDL-C) and elevated high sensitivity C-reactive protein (hs-CRP) than healthy control subjects [3]. Thus it is plausible that the accelerated CVD mortality among AD may be the result of several coexisting abnormal CVD risk factors. AD per se may have contributed to the excess mortality, although inadequate or supra-physiological glucocorticoid (GC) replacement therapy may have conferred adverse effects on CVD risk factors [4]. On the other hand, Norwegian patients with AD younger than 40 years, particularly males, were at risk for premature death due to infections, sudden death and acute adrenal failure [5].

Glucocorticoid (GC) excess has an important impact on cardiovascular risk factors [4]. In a large study of patients with hypopituitarism, a total daily replacement dose of hydrocortisone of ≥20 mg was associated with an increase in CVD risk factors, as evidenced by increased waist circumference, total cholesterol (TC) and low-density lipoprotein cholesterol (LDL-C) and triglycerides (TG) [6].

Unlike studies of hypopituitarism associated with secondary adrenal insufficiency, there is a paucity of studies evaluating the CVD risk in primary adrenal insufficiency. In a study of 38 AD patients, Giordano et al showed a higher proportion of patients who were hypercholesterolaemic and hypertriglyceridaemic than their matched controls [7]. Leelarathna and co-workers found a significant proportion of AD patients with dyslipidaemia [8], whereas Gurnell et al reported normal lipid profiles in 100 patients with AD [9]. Since the conventional GC replacement regimen often exceeds the normal endogenous cortisol production rate, it could be expected that using supra-physiological doses of GCs may increase the prevalence of risk factors for CVD [10]. Indeed, cardiovascular risk factors in adrenal insufficiency have shown to improve with a more physiological circadian cortisol profile during hydrocortisone replacement therapy, suggesting that not only the actual daily dose and total exposure are important, but also the profile of exposure [11].

Animal studies have shown that supra-physiological doses of GCs may raise TG, TC, LDL-C and high-density lipoprotein cholesterol (HDL-C) concentrations [12], [13]. GCs reduce hepatic lipase activity, which reduces the metabolism of HDL2 to HDL3, resulting in increased HDL-C concentration. Apoprotein A1 has also been found to increase in response to GCs, resulting in increased HDL-C [14]. GCs have also been reported to reduce lipoprotein lipase activity resulting in an increase in TG [15]. Down regulation of LDL receptors may account for the rise in LDL and TC [16]. Furthermore, diseases coexisting with AD, such as hypothyroidism and diabetes mellitus (DM) could also contribute to alterations in lipid concentrations and increase in risk for CVD [17].

Lipid patterns differ amongst countries and changes in time have also been observed. Sweden and South Africa are developed and developing countries, respectively and therefore geographical regions may influence lipid profiles and blood pressure of AD patients. Studies from Sweden have shown a decrease in the prevalence of hypercholesterolaemia (total cholesterol and triglycerides) in the general population [18]–[20], but those with basic education and living in rural areas had higher risk of developing hypercholesterolemia and hypertriglyceridemia, compared to those with higher education and living in urban areas [20]. Reliable data reflecting CVD prevalence in Africa are scarce. Nevertheless, some data show an increase in the incidence of CVD in Africa [21], explained by traditional CVD risk factors [22].

Our previous studies and personal communication indicate that patients with AD are likely to be treated with lower doses of GCs in South Africa, compared with Sweden [11] [23]. As Swedish patients with AD have been shown to have double the relative risk of death, predominantly due to cardiovascular disease [1], [2], and South African patients have an adverse lipid profile we wished to compare cardiovascular risk factors in matched Swedish and South African AD patients for age, gender, ethnicity and body mass index to determine whether glucocorticoid dose and geographical areas play a role in influencing cardiovascular risk in these patients. The primary aim of this study was to compare plasma lipids in patients with AD in South Africa and Sweden. Secondary objectives were to compare markers of cardiovascular inflammation.

## Patients and Methods

The Research and Ethics committee of the University of Cape Town and local research ethics committees in South Africa approved this study. These were from the respective research and ethics committees overseeing the various faculties of health sciences including the Nelson Mandela School of Medicine, University of Kwazulu-Natal, University of Stellenbosch, University of the Free State, University of Pretoria and the University of Witwatersrand. The Regional Ethical Review Board at the University of Gothenburg approved the study in Sweden. All patients received written and oral information and gave written informed consent prior to being enrolled in the study.

### Patients

AD patients ≥18 years of age in the western part of Sweden were invited to participate. Enrolment took place in Gothenburg between 2005 and 2008. The South African AD patients were selected from the South African Addison's disease study database, a cohort which enrolled patients between 2005 and 2010, the results of which have been published previously [24]. After enrolling the Swedish patients, patients matched for age, gender, BMI and ethnicity from the South African database were included.

### Clinical and demographic data

The Swedish cohort included only white patients; hence the ethnicity was restricted to white patients in the South African cohort. Disease duration, history of CVD risk factors including hypertension, diabetes mellitus (DM), lipid-lowering therapy and smoking were recorded. The doses of hydrocortisone and fludrocortisone were documented, in addition confirmation that the doses had not been altered for at least three months, prior to being enrolled. Clinical examination included blood pressure, body mass index (BMI) and confirmation that they were clinically stable without a superimposed acute illness.

### Biochemical assays for lipids and markers of CVD

#### Lipid assays

Lipid assays were conducted in two separate laboratories; the Gothenburg subjects were analysed by the Division of Clinical Chemistry, Sahlgrenska University Hospital, Gothenburg, while the South African subjects' sera were analysed in the Division of Lipidology at the University of Cape Town. Both laboratories comply with Precinorm (Boehringer Mannheim GmbH, Mannheim, Germany), which has international consensus values for plasma lipids, ensuring that magnitude of the potential difference is acceptable at 10%. In the case of South African patients, the assays for TG and TC were performed using commercially available enzymatic kits, standard curves and calibrators. The respective kits for TG, TC, were KAT (Roodepoort, Gauteng, South Africa) and HDL-C was determined using the first step in the Gidez assay, which yields HDL-C in the supernatant of a heparin-Mn precipitation of apolipoprotein B-containing lipoproteins [25]. The inter-assay coefficient of variation (CV) for TC was 2.2% at 2.4 mmol/L and 1.8% at 4.99 mmol/L, for TG 2.8% at 1.2 mmol/L and 2.2% at 2.2 mmol/L, for HDL-C 2.3% at 1.4 mmol/L and 2.3% at 0.8 mmol/L and LDL-C was calculated by the Friedewald equation: LDL-C  =  TC – HDLC – (TG/2.18), provided TG <4.5 mmol/L [26] with a CV of 4.2%.

In the case of Swedish patients, TC, TG, HDL-C and LDL-C (LDL-C from October 2007, 7 patients) were determined by enzymatic techniques (Modular P800, Roche Diagnostics, GmbH, Mannheim, Germany). The within-assay CV for TC, TG, HDL-C and LDL-C determinations was 3% (at 4 and 6 mmol/L), 4% (at 1 and 2 mmol/L), 5% (at 1 and 2 mmol/L) and 4% (at 2 and 5 mmol/L) respectively. The LDL-C was calculated according to Friedewald's formula prior to October 2007 (45 patients) and if TG values <4.5 mmol/L [26].

#### Highly sensitive C-reactive protein (hs-CRP) and Adiponectin

For both the South African and Swedish patients, hs-CRP and adiponectin were measured in the same laboratory at the Division of Clinical Chemistry, Sahlgrenska University Hospital, Gothenburg.

hs-CRP was measured by using highly sensitive particle immuno-turbidometric assay (Roche Diagnostics, GmbH, Mannheim, Germany), with a CV of 4% and 3% at serum concentrations of 1 and 15 mg/L, respectively. hs-CRP was corrected for SA patients after excluding a single outlier of 77 mg/L from the analysis. Adiponectin was measured by using ELISA kit (Millepore Corp., Billerica, MA, USA) with a CV of 11% at 2.1 mg/L and 7% at both 11.5 mg/L and 22 mg/L.

The thyroid stimulation hormone (TSH) was measured in Sweden for the Swedish patients, using the Roche Modular/Cobas (Rotkreuz, Switzerland) analyser and in South Africa, the Abbott Architect (Illinois USA) for the SA patients was used. The reference intervals for TSH in the South African and Swedish laboratories were 0.35–4.94 mIU/L and 0.27–4.2 mIU/L, respectively.

#### Statistical methods

The Shapiro-Wilk test was used to determine the distribution of each variable and the chi-squared test was used to assess significance for proportions. Normally distributed variables were compared using independent t-tests. Assessment for significant differences in non-parametric data was performed using the Mann-Whitney test. Statistical significance was reached when the *p-*value <0.05. 95% confidence intervals were constructed using the Wald approximation. Statistical software IBM® SPSS® 17.0, Somers, NY 10589 USA was used.

## Results

The Swedish and South African patients were well-matched with respect to age and BMI (Table 1). Both cohorts were white. Total daily hydrocortisone dose was higher among the Swedish patients (33 mg, range 10–50) than the South African patients (24.3 mg, range 5–40); p<0.001. Patient characteristics are demonstrated in Table 1.

**Table 1:** Clinical characteristics of patients with Addison's disease from Sweden (SE) and South Africa (SA).

Characteristics	SE	SA	*p*-value
N	55	55	
Age (SD) years*	52.9 (13.0)	52.6 (14.4)	0.897
Female N(%)	36 (65.5)	36 (65.5)	-
BMI (SD) kg/m^2*^	25.3 (3.2)	25.8 (4.1)	0.414
Hypertension N(%)	10 (18.2)	12 (21.8)	0.565
Diabetes N(%)	10(18.2)	12 (21.8)	0.599
Lipid lowering therapy N(%)	9 (16.4)	11 (20)	0.757
Smokers N(%)	4 (7.3)	4 (7.3)	0.632
Duration of disease (SD) years^*^	17.3 (11.3)	14.2 (12.0)	0.173
TSH mU/L *	2.57 (2.5)	1.97 (2.7)	#
Hydrocortisone (SD) mg^*^	33.0 (8.14)	24.3 (8.0)	0.001

Data presented as: * mean (standard deviation), N: number.

#: Not done as the method and reference ranges differ.

All the patients in the South African cohort were assessed for 21-hydroxylase autoantibodies with the majority of patients (76%) being positive, 1 patient had tuberculosis and the remainder were thought to have idiopathic AD. In the Swedish cohort, 73% were tested for 21-hydoxylase autoantibodies of whom, 83% had positive autoantibodies, and in the remainder the aetiology was idiopathic. Approximately 20% of the patients in both cohorts had hypertension and diabetes mellitus.

### Lipids, hs-CRP and adiponectin

**Figure 1:**
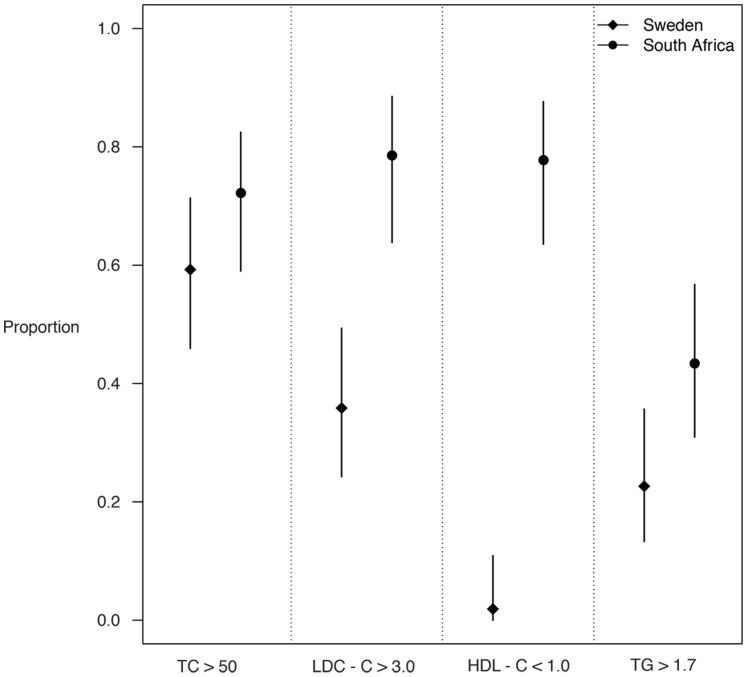
Bar chart showing the comparative proportions and 95% confidence intervals of Swedish and South African patients exceeding the recommended targets for TC <5.0 mmol/L; (p = 0.160), LDL-C <3.0 mmol/L; (p<0.0001), TG <1.7 mmol/L; (p = 0.02) and failing to achieve an HDL-C >1.0 mmol/L; (p<0.0001), according to ATP III NCEP (Adult Treatment Panel III, National Cholesterol Education Programme) recommendations.

The concentrations of TC, TG and LDL-C were lower, but HDL-C was higher, in the Swedish patients, compared with the South African patients. The hs-CRP was reduced in the Swedish group, compared to the South-African patients (Table 2). Adiponectin did not differ between the cohorts. The differences in lipids, lipoproteins and hs-CRP remained even after removing patients on lipid-lowering therapy and patients with diabetes mellitus from the statistical analysis (data not shown). A large proportion of the South African patients and to a lesser extent the Swedish patients, had unfavourable lipid levels exceeding the recommended targets defined by the NCEP ATP III guidelines (Figure 1) [27]. The relative proportions of Swedish patients, compared with South African patients exceeding a TC >5.0 mmol/L, LDL-C >3.0 mmol/L, TG >1.7 mmol/L and a HDL-C <1.0 mmol/L were 59.3% vs. 72.2%; (p = 0.160), 35.8 vs. 78.6%; (p<0.0001), 22.6% vs. 43.4%; (p = 0.02) and 1.9% vs. 77.8%; (p<0.0001), respectively.

**Table 2:** Serum lipids, inflammatory markers and adiponectin in patients with Addison's disease from Sweden (SE) and South Africa (SA).

	SE	SA	p-value
N	55	55	
TG (IQR) mmol/L^Φ^	0.96 (0.74–1.6)	1.59 (1.1–2.46)	<0.001
TC (SD) mmol/L^*^	5.13 (0.87)	6.02 (1.50)	<0.001
HDL-C (SD) mmol/L^*^	1.86 (0.46)	0.80 (0.40)	<0.001
LDL-C (SD) mmol/L*	2.75 (0.80)	4.43 (1.44)	<0.001
hs-CRP (IQR) mg/L^Φ^	0.99 (0.57–2.10)	2.15 (0.93–5.45)	0.003
Adiponectin (IQR) mg/L^Φ^	12.60 (8.18–17.9)	13.50 (8.10–19.6)	0.34

Data presented as: ^*^ mean (standard deviation) ^Φ^ median (interquartile range).

## Discussion

South African AD patients exhibit a more atherogenic profile with higher LDL-C, TG, and lower HDL-C, and elevated hs-CRP concentrations, despite being exposed to lower daily hydrocortisone doses, in comparison with Swedish AD patients. Our findings collectively suggest that the CVD risk is markedly elevated, as reported with AD [1], albeit that South African AD patients on lower doses of hydrocortisone, have greater risk than Swedish AD patients.

While the increased HDL-C among Swedish patients could be explained on the basis of an elevated cortisol exposure [14], other health promoting factors in the Swedish population could be accounting for this. Poor lifestyle may significantly contribute to dyslipidaemia and cardiovascular risk among South Africans. Environmental and economic factors can impact lipids with Reddy et al, predicting a rise of early-onset vascular events and the burden of CVD in the Third World [28]. The Scandinavian countries by comparison, have enjoyed long-standing emphasis on reducing cardiovascular risk factors. The 4S study has been pivotal in raising awareness on detection of lipid abnormalities in Northern Europe [29] and could have influenced the Swedish population favourably. It is conceivable that in First World countries, greater willingness exists among the public to pursue physical activity and healthier food options.

Neither ethnicity, nor BMI differed between these two groups of AD patients due to the matching of the two groups. The patients demonstrated an increased weight in both groups, but data on waist circumference, dietary habits, alcohol consumption and of physical activity were not available. The extent, to which life-style, socio-economic, educational attainment and levels of health-care factors contributed to these differences found between the cohorts, is uncertain.

Both uncontrolled diabetes and hypothyroidism are known to adversely influence the blood lipid profile, but the differences between the cohorts in lipid profiles persisted, even after exclusion of patients with diabetes mellitus. Moreover, the majority of the Swedish and South African patients were euthyroid. Although the lipid profile was more favourable in the Swedish group, the TSH was numerically higher than the South African group, indicating the difference in lipids and lipoproteins between the groups is not explained by the difference in thyroid functions.

There could also be some genetic predisposition, either a favourable or unfavourable variation accounting for the differences between these two distinct geographical groups. Genetic defects for example, Apo E2/E2 status, which is known to occur in 1:50 of the general population, can produce elevated TG levels [30]. Genetic deficiencies of lipoprotein lipase may also produce elevated TG levels, especially with excess hydrocortisone replacement [31]. Our respective cohorts were not screened for these or other genetic defects and are probably too small for genetic variants to play a significant role.

hs-CRP is an acute phase reactant protein that is associated with ischaemic vascular disease [32]. The influence of dehydroepiandrosterone (DHEA) on hs-CRP in adrenal insufficiency has been evaluated, but no change was found when DHEA was substituted, albeit that the mean hs-CRP was normal at 1.4 mg/L [33]. In addition our cohorts were exclusively white, eliminating ethnicity as a cause for the elevated hs-CRP.

It is intriguing that Nazmi et al, showed that poverty and socio-economic factors may also account for a raised hs-CRP [34], and it is entirely plausible that the lower socio- economic status that exists in South Africa may contribute to the relative increase, compared with the Swedish AD patients. Another possible explanation for comparatively reduced hs-CRP in the Swedish group is their higher hydrocortisone substitution doses as Short term GC treatment has been shown to decrease hs-CRP [35]. hs-CRP has also been shown to associate with abdominal obesity and it is possible that the higher concentrations in the South African cohort indicate that they harbour relatively more visceral fat with insulin resistance and impaired glucose metabolism, but this is not yet been tested. The similar concentrations of adiponectin in both groups however, do not support a difference in insulin sensitivity.

Although CRP is reduced by statins, the difference in hs-CRP in the 2 cohorts remained significant when those receiving lipid-lowering therapy were excluded. The markedly raised hs-CRP in the South African cohort is of concern and should be an important impetus to modify, CVD risk factors aggressively in this group.

There is no consensus on the appropriate daily hydrocortisone doses in AD patients. Based on the daily cortisol production rate in healthy adults and the fact that doses below 20 mg per day have approximately 90% bioavailability, current doses can be considered too high in most patients. Doses above or equal to 20 mg have been associated with adverse metabolic outcome in hypopituitary patients [36]. There may be a negative association between dose exposure and bone mineral density [37] and lower doses may be associated with better health related quality of life [6], [38], [39]. Chronic over-replacement with GCs could have influenced the cardiovascular profile in the Swedish patients and the non-physiological replacement therapy may have potentially influenced both groups negatively. It is conceivable that the differences in lipid profiles may have been even greater had the hydrocortisone replacement been lower in the Swedish AD patients.

This study has a number of limitations. The study was designed to include fasted participants with their hydrocortisone tablet taken prior to sampling, but this could not be verified in the South African cohort. It is possible that the TG concentrations in the South African cohort may have been taken in the post-prandial state, accounting for the relatively higher TG concentrations. Assessment of glucose tolerance in relation to hydrocortisone doses, by performing oral glucose tolerance tests may have provided insights into the aetiology of the relatively high TG concentrations in both cohorts. Anthropometric measurements such as waist circumference to estimate the visceral fat could have complemented our understanding of the hypertriglyceridaemia. Important lifestyle factors such as diet, alcohol consumption and physical activity were not evaluated in this study. Moreover, the interpretation of the data would have been strengthened by matched healthy controls for both cohorts to determine the extent to which patients differed from their background populations. Despite our observed adverse lipid profiles and cardiovascular risk factors particularly in the South African cohort, it remains uncertain as to whether these are due to AD per se or its management.

While we have identified that South African patients, compared with Swedes with AD have higher risk using cardiovascular risk factors as a crude assessment, accurate mortality data in South Africa unlike Sweden would be very difficult to generate. This study should prompt further research into the genetic profiles of these patients, in order to explain our observed vast differences in cardiovascular risk factors in these cohorts.

## Conclusions

Both groups of patients with AD have increased CVD risk, but the South African group exhibits a more atherogenic profile with higher TC, LDL-C, hs-CRP and lower HDL-C compared with the Swedish group, despite the former patient group being replaced on lower doses of hydrocortisone. Although the higher doses of hydrocortisone replacement in the Swedish group could have aggravated the CVD risk profile, the relatively lower doses of hydrocortisone replacement in the South African patient group did not appear to mitigate against CVD risk factors. Environmental factors are likely more important in modifying CVD risk factors associated with AD than hydrocortisone dose. Nevertheless, we believe it is critical to treat hypertension, diabetes and lipid abnormalities, while encouraging smoking cessation in this group of AD patients where cardiovascular risk factors are prevalent. Overall, our analysis reveals that despite higher doses of hydrocortisone replacement, the cardiovascular profile in Sweden is far more favourable than in South Africa, where lower doses of hydrocortisone are used.
